# Hospitals from the Patients' Point of View

**Published:** 1914-06-20

**Authors:** 


### HOSPITALS FROM THE PATIENTS' POINT OF VIEW.

(Contributions to this Section are Invited.)

### Three Weeks in a Pay-Ward.

Some time ago I had to undergo a serious abdominal operation, and the doctor recommended me to apply for admission in the pay-ward of a certain well-known institution which makes a practice of receiving women, chiefly for operations, and, being partially endowed, is enabled to offer them terms at which it would be quite impossible for any commercial nursing home to accept patients, especially if there are shareholders in the background expecting interest on their capital. As my husband is a man of only moderate means I was only too thankful to take advantage of my doctor's letter of recommendation, which forms the preliminary step towards admission into the pay-ward. I was, I may admit frankly, somewhat horrified at the various forms which were thereupon despatched for my husband and myself to sign, and there was a certain grimness in the undertaking that he had to make to be responsible for my removal "alive or otherwise." How often a euphemism is worse than the naked truth! It was, however, a relief to find that, once these forms with their answers to questions were filled in, I was spared the unpleasantness of being cross-examined upon them. I have always had such a horror of "C.O.S." methods that I entered the institution with a comparatively light heart, on receiving, without further trouble, an intimation that I was expected on such-and-such a day. This, as a matter of fact, I was unable to take advantage of, since, as the sister subsequently informed me, the anxiety attendant upon a decision to be operated upon often causes, as it did in my case, a temporary inhibition of natural functions which only waiting can cure.

Well, the day came, and, bringing one trunk as requested, I arrived in company with my husband after tea. I felt very miserable when he left, though a kind friend had sent some flowers to greet me on my arrival; but I did not like the probationer who was the first person whom I saw. She was fussy and unskilful, and was always knocking things over. When she told me that she had only been in the place some weeks, I could not help wondering why such untrained individuals were permitted in that part of an institution reserved for those who were contributing, as I was, between two and three pounds a week towards the cost of their care.

I unpacked, had a fragment of supper, for my appetite vanished with my husband, and I got into bed. Then arrived the nurse, who, I was happy to see, was at least trained; and then came the inevitable large dose of medicine. I expected this and do not complain of it, nor of the wretched night I had on account of the noise. As soon as the wheeled traffic ceased the people in the neighbourhood, a very poor one, began to make a night of it. Quarrels, laughter, drunken singing were constant, and if I could have slept, which I admit is doubtful, these would have kept me awake. I suppose I shall be told that the expense is prohibitive, but if pay-wards cannot be built in the quieter streets of London, because even quiet has a rental value in cities, why, at least, cannot double windows be fitted? It is customary everywhere abroad, and the ventilation difficulty could be overcome, at least during the night, by fans or similar contrivances. In the interests of other patients I throw out this suggestion, and hope that hospital committees may at least consider whether there is no possibility of carrying it out.

The next morning I had an opportunity of testing the efficacy of my electric bell, which was conveniently hooked on to my bed in case of need. The bell certainly rang; as I had to ring it six times in succession I have a vivid memory of the fact. At last the nurse arrived, and when I told her I could not drink castor oil at night without urgently requiring to take the earliest possible advantage of it in the morning, she replied that everyone was using bed-pans at this hour; that the supply was deficient; and that there were not enough nurses to attend to the patients in this part of the building, a fact which was apparent from the constant ringing that went on while she was speaking, and of which for the time being she took no notice whatever.

Hardly was this problem at last surmounted, for me at least, than another nurse arrived and told me that she had orders to shave me for the operation. In my ignorance I had no idea that it would be necessary, and only endured the process by thinking that after all I might be dead before night! The operation was to take place in the afternoon. At last the shaving was over, and I lay back wondering what next was in store. The rest of the morning dragged extremely, and was made no more pleasant by the noisy laughter of the nurses and the banging of the doors which was I will not say constant, that would be an exaggeration, but at least frequent. As I was next but one to the nurses' room perhaps I heard more than anyone except my intervening neighbour. But I may say at once that during the three weeks of my stay there was a great deal of chattering, laughter, and banging of doors on the part of the nurses which surely was quite unnecessary.

On my sensations on going up to the operation theatre I need not dwell. I had been warned by a friend not to wear any nightdress the future of which I valued, and so the flannel garment which was provided, and the long stockings, came as a relief as well as a surprise. The chloroform, or whatever it was, was given in a separate room next to the theatre, and my personal misery did not begin until I recovered several hours later (for the operation lasted an hour and I was a long time coming round) in my own room again. Soon after I awoke, with the agonising sickness of one who had a large abdominal wound; they gave me an injection of something, so that I was largely unconscious while the sickness was proceeding, and the nurses were both skilful and kind in holding me in such a way as to make the "strain" less severe. The first day and the second passed without much to describe—I was only beginning to have "a point of view" at all, and rather startled at the symptom—but again I had to wait and ring, and ring and wait before I could get attended to.

This was really the main charge that I bring against the institution and one which I state the more frankly in that I gladly admit that were I ever to repeat the awful experience—for a severe surgical operation is, in point of fact, just that and no less—I would go again to this place to have it done. The nurses individually were, at least on my floor, attentive and kind; the sister was also quiet and therefore comforting to a sick person; but they could not or would not answer the bells with anything like promptitude. I believe that an extra nurse would have made some difference; I am sure that a stricter discipline would have made more. This brings me back to my friend the "flighty" probationer. She would have been more under control had she had a more constant supply of work; but in the intervals that are bound to occur she laughed and chatted and pushed about the passages in a way that as a symptom of health was delightful, but which was unsuitable in the vicinity of sick persons. I think, if I may venture the criticism, that she would have gained more in a general ward, where she would always have been under the eyes of others; and, much as I liked her (when I got better), I cannot but think the authorities made a mistake in having her in the private rooms of the institution.

The morning was certainly the least happy part of the day. One was awakened by the night nurse having to make the beds before going off duty, at 6 A.M.; and then a little later, and every day, the unmentionable discomfort of ringing one's bell helplessly, only to be told, after an interval of sometimes a quarter of an hour: "All the bedpans are in use." That, surely, is faulty administration? Why could not one be assigned to each room? And why, too, at this, the regularly busiest time of every day, could not the staff have been increased or co-ordinated so that there was no chance of bells being rung in vain? To have achieved this is only a matter of arrangement. How it is to be done is the matron's business to decide.

I would not close this haphazard account of my sensations and ideas on a note of discord. So let me in fairness and gratitude point out the features which impressed me as praiseworthy. First of all, the food was excellent, and had the two primary virtues of sick-room cooking that it was very punctual in time and always served steaming hot. This was daily something to be very thankful for. Though it is not possible to ring many changes on milk puddings and suchlike dishes, all were daintily served; the crockery unbroken, the linen very clean, both of which are signs of good management. As to the nurses, they were uniformly kind, and would have been beyond criticism had there been sufficient administrative control to check their natural appetite for conversation in the passages. There were, in my opinion, too few of them on duty in the morning, and the evil consequence of this was that they were uniformly somewhat careless of answering bells at any time. When the door of my room was open I could see them standing outside the lift, and often observed them finishing a conversation before attempting to answer a bell that was continuing to ring. These faults were faults of administration, and not so much faults in the nurses themselves. As women, they were nice-mannered to the patients and skilful.

In conclusion, I think that there should have been appliances on the doors to have prevented them from banging, that double windows ought to have been installed, and, finally, that the flower vases might sometimes have been washed with soap-suds or strong soda, to remove from them the accumulated deposits of many months, perhaps of years. The servants were extremely quiet and quick, and I think that in these two respects they had something to teach the nurses. The institution itself was beautifully kept, and I shall always be grateful for the absence of red tape in dealing with applications for admission. But if this article ever falls under the eye of any of the staff I hope that they will realise, first, that I am writing in a friendly spirit; and, secondly, that if I have touched on small points in my criticism, these small points make all the difference where a high standard is involved. "The little more, and how much it is; the little less, and what worlds away." That is the Patient's Point of View in a nutshell.

